# Second-Hand Smoke and Its Synergistic Effect with a Body-Mass Index of >24.9 kg/m^2^ Increase the Risk of Gout Arthritis in Indonesia

**DOI:** 10.3390/ijerph18084324

**Published:** 2021-04-19

**Authors:** Maria Dyah Kurniasari, Ferry Fredy Karwur, Rosiana Eva Rayanti, Edi Dharmana, Yohanes Andy Rias, Kuei Ru Chou, Hsiu-Ting Tsai

**Affiliations:** 1 School of Nursing, College of Nursing, Taipei Medical University, Wu-Xing Street, No 250, Taipei City 11031, Taiwan; maria.dyah@staff.uksw.edu (M.D.K.); kueiru@tmu.edu.tw (K.R.C.); 2 Department of Nursing, Faculty of Medicine and Health Sciences, Universitas Kristen Satya Wacana, Diponegoro Street, No 52-60, Salatiga City 50711, Indonesia; Rosiana.evarayanti@uksw.edu; 3 Department of Nutrition, Faculty of Medicine and Health Sciences, Universitas Kristen Satya Wacana, Diponegoro Street, No 52-60, Salatiga City 50711, Indonesia; ferry.karwur@uksw.edu; 4 Faculty of Medicine, Universitas Diponegoro, Prof. Sudarto Street, No.13, Semarang City 50275, Indonesia; edidharmana@yahoo.com; 5 Faculty of Health and Medicine, Institut Ilmu Kesehatan Bhakti Wiyata Kediri, College of Nursing, KH Wachid Hasyim Street, No.65, Kediri City 64114, Indonesia; yohanes.andi@iik.ac.id; 6 Center for Nursing and Healthcare Research in Clinical Practice Application, Wan Fang Hospital, Taipei Medical University, Taipei 11696, Taiwan; 7 Department of Nursing, Taipei Medical University-Shuang Ho Hospital, Taipei 23561, Taiwan; 8 Psychiatric Research Center, Taipei Medical University Hospital, Taipei 11031, Taiwan; 9 Post-Baccalaureate Program in Nursing, College of Nursing, Taipei Medical University, Wu-Xing Street, No 250, Taipei 11031, Taiwan

**Keywords:** second-hand smoke, synergistic effect, body-mass index, gouty arthritis, Indonesian

## Abstract

To analyze the association between smoking status (active smoking and exposure to Second-Hand Smoking (SHS)) and the synergistic effect of smoking status and BMI with gout risk, a community-based case-control design was undertaken among 385 participants, including 304 healthy controls and 81 gout patients from seven community health services. Adjusted Odd Ratios (AORs) and 95% Confidence Interval (CIs) of gout for active smoking and SHS were 3.26 (95% CI = 1.07~9.90) and 4.67 (95% CI = 2.18~10.00) compared to non-smokers. Time-dependent manner of active smoking and SHS significantly increased gout risk with AORs and 95% CIs of 5.95 (1.41~25.03) and 10.12 (3.51~29.14). Dose-dependency of active smokers and SHS showed AORs and 95% CIs of 5.15 (1.28~20.63) and 4.37 (1.33~14.28). Smoking 20 cigarettes (one pack) per day for one year is equivalent to one pack-year. Active smoking >20 pack-year and SHS > 26.5 pack-year increased gout risk with AORs and 95% CIs of 7.18 (1.53~33.67) and 9.95 (3.64~27.22). Participants who smoked (active smoking and SHS) and with Body Mass Index (BMI) of > 24.9 kg/m^2^ synergistically increased gout risk, with an AOR of 9.65 and 95% CI of 3.25~28.65, compared to BMI ≤ 24.9 kg/m^2^ and non-smoker. Smoking status (active smoking and SHS) and the synergistic effect of smoking status and BMI increased gout risk in Indonesia.

## 1. Introduction

Gout is a type of autoinflammatory arthritis that is found worldwide [1]. Globally, as a burden related disability [2,3], the prevalence and incidence of gout has increased over the past 50 years [4]. Nonetheless a limited number of studies have investigated the distribution and time pattern of this disease internationally, regionally and nationally [2,5]. The number of prevalent cases, incident cases and deaths have also increased over time in the United States [6,7], Italy, South Korea, Australia, New Zealand, and Taiwan [4]. An escalating number of gout cases was also found in developing countries, and of these countries Indonesia continues to have the highest prevalence of gout, with the disease affecting 1.7% of adults [4]. A study also indicated that gout was a prevalent arthritic disease in the Malayo-Polynesian ethnic groups [8]. In the population of Malayo-Polynesian ethnic groups, gout is the third most common type of arthritis in Indonesia [9], and the highest prevalence is in North Celebes [10]. 

A survey in Makassar, South Celebes, and a study in Bandungan, Java, Indonesia presented a higher prevalence of gout and hyperuricemia than in Caucasians [8,11]. Interestingly, a study in Indonesia reported that, of the total of hyperuricemia patients, 65.6% were under 40 years old. This indicated that hyperuricemia occurred among a young population [12]. A prior study which recruited patients with chronic gout and age- and sex-matched healthy controls derived from 28 community health centers in Indonesia reported that 86.3% of gout patients had renal impairment, and only 7.4% of healthy controls [8]. Gout patients often delay getting efficacious treatment, which generates chronic tophaceous gout with irreversible disabilities and deformities. More than 50% of gout patients delay getting efficacious treatment and have been screened for tophi 7~9 years before access to treatment [8]. This evidence confirmed limited gout management in Indonesia in recent years [13]. These empirical issues indicate that it is vital to investigate gout-related risk factors to protect Indonesians from this disease.

The multiple risk factors of gout are an interplay between genetic and environmental factors [14]. As reversible factors, environmental factors are important issues to raise in prevention strategies against gout, including hyperuricemia [15], a high body-mass index (BMI) [16,17], high blood pressure [18], and alcohol consumption [19,20]. Numerous studies have postulated that smoking can increase oxidative stress, urate levels [21,22], and gout [23,24]. Additionally, a single cigarette immediately lowers plasma serum antioxidant levels that can consistently damage endothelial cells [25]. In contrast, a study in Framingham established a decrease of 20%–30% in gout cases linked to cigarette smoking [24]. The association between smoking and lower risk of gout has also been reported in another previous study [14]. Active smoking has also been significantly associated with lower serum urate concentrations [10,24,25,26,27]. Besides, a study conducted in Singapore conveyed that participants who are active smokers lower by 27% the risk of gout incidence [26]. However, this previous result was only found among male participants [26]. The association of smoking status and gout remains controversial, and this issue has never been investigated in Indonesia. Moreover, Indonesia is recognized as one of the top five countries in the world for tobacco use. Due to its large population base, it is a key destination for the expansion of tobacco companies, with minimal control of tobacco production. Smoking habits are initiated among males at the early age of 15–20. Early smoking habits also arose among females [28]. Therefore, estimating the relationship between active smoking and gout among Indonesians is necessary.

Remarkably, in terms of the smoke inhalation and exhalation pathway, compares to active smoking, SHS has a relatively shorter duration to manifest cardiovascular diseases [29]. The particles in SHS are smaller than in an active smoker, since they are generated in an oxygen-poor environment at lower temperatures and contain high concentrations of a multitude of toxic agents such as ammonia, nitric oxides, and carcinogens [30]. Besides, a previous interventional study revealed that there was no effect of second-hand smoke (passive smoking) on serum urate. Nonetheless, this result was exhibited among a small sample size [31,32]. On the other hand, SHS was proven to produce inflammation through increased levels of inflammatory cytokine interleukin (IL)-6, with an adjusted odds ratio (AOR) of 1.10 after adjusting for covariates compared to participants who are unexposed [33,34]. It was suggested that SHS stimulates the release of norepinephrine, which can stimulate IL-6 synthesis in the liver and spleen [34]. Meanwhile, gout is an autoinflammatory disease activated through the proinflammatory IL-6 cytokine [18]. This study indicated that the IL-6 inflammatory cytokine was positively associated with inflammatory activity in gout. We, therefore, hypothesized that SHS stimulates an increased inflammatory cytokine IL-6 response and might be a potential risk factor for gout. Nevertheless, controversial findings have been conveyed by a previous study regarding the relationship between second-hand smoking and urate as well as second-hand smoking and inflammation. Likewise, no study has been conducted on the relationship between SHS and gout. There is a critical gap in the knowledge of the role of SHS in gout risk. 

A higher BMI was also proven to be a critical reversible risk factor for gout [16,35]. This was strongly sanctioned due to a reduction in renal clearance rates for urate among participants with a high BMI [35]. Interestingly, a previous study revealed that excessive adipose tissues at a BMI of >24.9 kg/m^2^ combined with smoking habits initiated proinflammatory cytokines that increase ROS (Reactive Oxygen Species) as the essential signaling molecules that contribute to the development of inflammatory diseases [27,36] and nitrogen species by macrophages and monocytes [27]. Besides, it induces systemic inflammation and, consequently, develops into chronic inflammation. Hence, it was suggested that urate clearance was impaired [27,37] and caused hyperuricemia [38].

Remarkably, smoking and obesity have become public health problems that will likely persist for decades to come [39], including in Indonesia. The prevalence of smoking in adolescents (10–18 years of age) has continued to rise from 7.2% in 2013 to 8.8% in 2016 and 9.1% in 2018. Moreover, the prevalence of SHS exposure among Indonesians reached 96.9 million [10]. The percentage of obesity in Indonesia is escalating rapidly from 20.7% in 2016 to 33.5% in 2019 in both sexes [26] and all age groups [40]. Some 13.5% and 28.7% of Indonesians aged ≥18 years are overweight and obese, respectively [41]. We hypothesized that both active smoking and SHS, along with their synergistic effect with a high BMI, play essential roles in gout among Indonesians. However, these issues of active smoking, exposure to SHS, and BMI in relation to gout risk have never been clarified in Indonesia; in particular, the relationship between SHS and gout risk has never been estimated worldwide. To address these needs, this study analyzed the association between smoking status (active smoking and exposure to SHS) and the synergistic effect of smoking status and BMI on gout risk in Indonesia. We hypothesized that smoking status (active smoking and exposure to SHS) and the synergistic effect of smoking status and BMI significantly increased gout risk.

## 2. Materials and Methods

### 2.1. Study Design

This study is a community-based retrospective case-control study to identify smoking status (active smoking and exposure to SHS) and the synergistic effect of smoking status and BMI on the gout risk in Indonesia. 

### 2.2. Participants

G-Power 3.1 software was used to calculate the sample size. Considering potential confounding factors, an alpha level (α) = 0.007, a power (1 − ß) of 0.97 [42], and 1.20 [24] as risk probability, the ratio of gout patients to healthy controls was 1:4, and therefore we needed a sample size of 360. To maximize the power of the study and minimize the covariance of variable as well as margin error, we therefore considered adding up to 10% more participants to the total sample, thereby increasing the sample size to 385. Thus, we enrolled 81 patients with gout and 304 healthy control participants.

The study was conducted in North Celebes, Indonesia, between July 1 and 31 August 2019. The study participants were recruited using stratified multistage cluster sampling. In the first stage, we conveniently selected North Celebes as one of the provinces in Indonesia which had the highest prevalence of gout and stratified it into 15 regions. In the second stage, two regions, including urban and rural areas, were randomly selected. In the third stage, only the urban area was chosen to participate in this research collaboration, which was Tomohon City. Seven community health service centers in Tomohon City were randomly selected. In the last stage, we recruited the eligible participants based on the study criteria. The inclusion criteria included (1) participants were Indonesian nationals aged ≥18 years, (2) diagnosed with gout within one year based on criteria guidelines for the diagnosis of gout from the Indonesian Rheumatologist Association [43], based on the American College of Rheumatology/European League Against Rheumatism (i.e., at least one episode of peripheral joint or bursal swelling, pain or tenderness, presence of MSU crystals in a symptomatic joint/bursa (i.e., synovial fluid) or tophus in the laboratory and imaging analysis) [44]. A visit to an outpatient clinic of the Public Community Health Service of Tomohon City was made by (3), and (4) agreed to participate in the study. The exclusion criteria were participants who recorded the presence of other types of inflammatory arthritis, including rheumatoid arthritis (RA) or spondylarthritis.

The inclusion criteria of healthy participants were (1) no history of gouty arthritis or other chronic diseases, (2) Indonesian nationals aged ≥18 years, and (3) who had agreed to participate in the study; these were employed as the control group. The exclusion criteria were participants suffering from any chronic diseases. 

**Figure 1:**
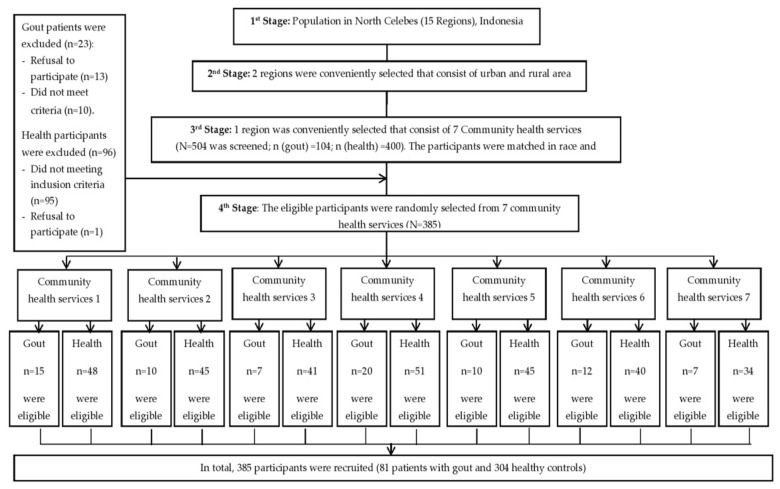
Participants Recruitment Flow Chart.

Four trained professional nurses collected all of the data. The data source of patient diagnoses was their medical records in the Public Community Health Service of Tomohon City. A flow chart of participant recruitment is shown in Figure 1.

### 2.3. Data Collection

#### 2.3.1. Physical Examination and Blood Biochemistry Analysis

A physical examination was conducted for blood pressure, and the BMI was assessed in a standardized medical examination. BMI was identified by measuring weight and height and confirmed using medical records. The body weight (kg)/height^2^ (m^2^) formula was utilized to calculate BMI [45]. Urate level measurement was obtained from the capillary blood samples by a fingertip puncture [46].

#### 2.3.2. Questionnaire

Demographic data were collected using a self-designed questionnaire survey containing participant’s demographic characteristics, including gender, marital status, educational level, income, occupation, and age [47].

Smoking status was assessed based on smoking habits or smoking history before the diagnosis. Smoking status, including active smoking, SHS exposure history, and the duration and dosage of smoking, was determined using a smoking status questionnaire survey, and the validity and reliability of the questionnaire were reported in a previous study [47]. An active smoker was defined as a participant who smoked more than one cigarette a day for at least one year. We also measured the total duration of smoking (in years) as well as the average number of cigarettes per day. If a study participant was not an active smoker, we further determined that participant’s exposure to SHS or passive smoke. SHS was defined as non-smokers exposed to the smoke of more than one cigarette per day for at least 15 min daily [47,48]. In our study, at least one of family member smoked in the home and was living in the same house, or the non-smokers were exposed to at least one active smoker in public places such as workplace, offices, restaurants, shopping centers, public transportation, cars, parks, schools, and daycare centers, etc., at a distance of less than 2 m; and conformation to the above definition of SHS was included to estimate SHS status [47,49,50,51]. 

If participants had been exposed to SHS, detailed questions were asked, encompassing the average number of cigarettes exposed to per day and the total exposure period (in years) [47]. A former smoker was classified as a smoker who had stopped smoking for one year or more during the interview [47]. Number of pack-years was calculated by multiplying the number of packs of cigarettes smoked per day by the number of years the person had smoked. For instance, smoking 20 cigarettes (1 pack) a day for 1 year is equivalent to 1 pack a year [52,53]. 

### 2.4. Ethical Considerations

The study protocol was reviewed and approved by the Institutional Review Board Ethics Committee (N201912052) of Taipei Medical University, Taiwan, and Universitas Kristen Satya Wacana, Salatiga, Indonesia (187/PE/KEPK.UKSW/2019), and conformed to the provisions of the *Declaration of Helsinki*. Written informed consent was obtained from each participant after they had received both verbal and written information about the research.

### 2.5. Data Analysis

A *X*^2^ test was used to examine the distributions of participant characteristics. Continuous data such as urate levels, blood pressure, duration of smoking, and the number of cigarettes consumed per day were categorized based on the mean if the data were normally distributed; while, if the distribution was not normally distributed, the data would be categorized based on median number. However, since the data on urate levels, blood pressure, duration of smoking, and the number of cigarettes consumed per day were not normally distributed, those data were categorized based on the median as the cut-off points [54]. Covariates in the final models were those that were significant or marginally significant in the univariate analysis such as gender, education level, age, urate, systolic blood pressure, BMI, and alcohol consumption. The unadjusted and adjusted odds ratios (ORs; AORs) with 95% confidence intervals (CIs) of the association between gout and risk factors, including smoking status (active smoking and exposure to SHS), duration (in years), number of cigarettes per day, the combination of dose and time for active and SHS exposure, pack-years of active smoking and SHS exposure were estimated by a multivariate logistic regression, after controlling for other covariates such as gender, education level, age, urate, systolic blood pressure, BMI and alcohol consumption. 

Then the synergistic effect between a BMI of >24.9 kg/m^2^ and being a smoker (active smoker or exposed to SHS) was estimated after creating four dummy variables for the following four (2 × 2) conditions: (1) BMI of ≤24.9 kg/m^2^ and a non-smoker (the reference condition or β00); (2) BMI of ≤24.9 kg/m^2^ and a smoker (active smoker or exposure to SHS) (β01); (3) BMI of >24.9 kg/m^2^ and a non-smoker (β10); and (4) BMI of >24.9 kg/m^2^ and a smoker (active smoker or exposure to SHS) (β11). We calculated the additive interaction or synergistic effect using the following categories: (1) if β11 = β01 + β10, no interaction; (2) if β11 > β01 + β10, a positive interaction revealing a synergistic effect as a result of more than additivity; and (3) if β11 < β01 + β10, a negative interaction [55,56]. *p* < 0.05 was considered statistically significant. The adjusted odds ratio with 95% confidence intervals (CIs) was applied to the association between a BMI of >24.9 kg/m^2^ and being a smoker (active smoker or exposed to SHS) and the risk of gout. We also controlled the covariates such as gender, education level, age, urate, systolic blood pressure, BMI, and alcohol consumption. All statistical analyses were performed using the Statistical Package for the Social Sciences (SPSS) vers. 25.0 (Chicago, IL, USA).

## 3. Results

### 3.1. Sociodemographic Characteristics and Determination of Risk Factors for Gout

From the total of participants, there were significant differences in distributions between patients with gout and healthy controls in gender, educational level, and age (Table 1). Significant differences were also found in the distributions of urate, systolic blood pressure, BMI, smoking status, duration of active smoking, duration of exposure to SHS, the number of cigarettes consumed per day for active smokers, and the number of cigarettes generating exposure to SHS (Table 2). Since the number of former smokers was very small, we therefore excluded former smokers in the analysis.

### 3.2. Association between Smoking Status and Gout Disease

The hypothesis of this study was supported by the results. Participants exposed to SHS and active smokers both had significantly increased risk of gout with respective AORs of 4.67-fold (2.18~10.00) and 3.26-fold (95% CI = 1.07~9.90) of developing gout compared to non-smokers after adjusting for confounding factors (Table 3). A higher risk of gout was found for active smokers who had smoked for more than 23 years (AOR = 5.95; 95% CI = 1.41~25.03) compared to non-smokers after adjusting for covariates (Table 3). It is noteworthy that an increased 10.12-fold risk (95% CI = (3.51~29.14) of gout was found in subjects exposed to SHS for more than 31.5 years compared to non-smokers after adjusting for covariates (Table 3).

Regarding the dose of cigarettes, an increased 5.15-fold risk (95% CI = 1.28~20.63) of gout was found in active smokers who smoked more than 16 cigarettes per day, after adjusting for covariates. Moreover, people exposed to ≤20 and more than 20 cigarettes per day (SHS) had an increased gout risk with respective AORs of 4.53-fold (95% CI = 1.89~10.84) and 4.37-fold risk (95% CI = 1.33~14.28) compared to non-smokers after controlling for confounding factors (Table 3).

The combinations of dose and time manner were also analyzed. The pack-year analysis shown that significantly increased risk of gout in both active smoking >20 pack-year and SHS exposure > 26.5 pack-year with the respectively AOR of 7.18 (95% CI 1.53~33.67) and 9.95 (95% CI 3.64~27.22; Table 3).

### 3.3. Synergistic Effects of BMI and Smoking Status on the Risk of Gout

Participants with both a BMI of ≤24.9 kg/m^2^ and smoking status had a significantly increased 3.18-fold (95% CI = 1.01~9.97) risk of gout compared to participants with both a BMI of ≤24.9 kg/m^2^ and being a non-smoker after adjusting for covariates. Moreover, a synergistic effect of a BMI of >24.9 kg/m^2^ and being a smoker for increasing gout risk was found, with an AOR of a 9.65-fold risk (95% CI = 3.25~28.65) after controlling for confounding factors compared to participants who both had a BMI of ≤24.9 kg/m^2^ and were a non-smoker (Table 4).

### 3.4. Association between Smoking Status and Hyperuricemia

After excluding four gout participants who underwent lowering urate therapy, the relationship between smoking status and hyperuricemia is shown in Table 5. Those exposed to SHS had significantly increased the risk of hyperuricemia, with AOR of 3.37-fold (95% CI = 1.93~5.89) for developing hyperuricemia compared to non-smokers after adjusting for confounding factor (Table 5). Active smokers also increased the risk of hyperuricemia with OR of 2.65 (95% CI = 1.53~4.59) compared to non-smokers although there was no significant difference after adjusting for confounding factors, with AOR 1.39 (95% CI = 0.63~3.07; Table 5).

## 4. Discussion

This study discovered some novel evidence that smoking status (active smoker and exposure to SHS) and the synergistic effect between smoking (active smoker or exposed to SHS) and a BMI of >24.9 kg/m^2^ significantly elevated the risk of gout. Interestingly, our study found that active smokers were more likely to have gout risk in time- and dose-dependent increases. The study results are consistent with previous research, which postulated that a smoking habit produces a 1.20-fold risk of gout [23,24]. In terms of the time- and dose-dependent characteristics, this study was consistent with a previous cohort study conducted between 1987–2013, which showed that current smoking led to = a 1.70-fold risk of developing gout [23]. A higher dose and prolonged duration of smoking showed a strong association with a systemic inflammatory response [57]. This phenomenon might be caused by an increase of free radical compounds in the smoke that stimulate oxidative stress production. Consequently, these can stimulate increased urate in the serum and IL-6 inflammatory cytokine, which is also positively associated with inflammatory activity in gout [18,57,58,59]. Moreover, avoidance of active smoking was recommended by the European League Against Rheumatism (EULAR) due to the increased cardiovascular risk associated with gout [60]. Therefore, the results of this study suggest that the intensity of cigarette smoke is a critical variable that can induce inflammatory response mechanisms among patients with gout.

However, the current study results were inconsistent with other prior studies, in which active smoking was found to reduce the risk of developing gout [61,62]. We suggest that the reasons for the different findings might be (1) diverse racial populations in each respective study; (2) not separating smoking status into active smokers and exposure to SHS; (3) a reduced risk of gout only being found in male smokers and not in female smokers; and (4) the difference in study design might also contribute since we assessed the association between active smokers and the risk of gout using a retrospective case-control study design. The result of the previous cohort study was based on limited access datasets from the Framingham Heart Study, which could have limited the validity for estimating the association [62]. Besides, (5) we suggested inconsistent results due to the smoking status categorization. A previous study reported that active smoking lowers gout risk; however, former smoking was included in the non-smoking group [62]. Meanwhile, our current study excluded former smoking in the final analysis. Another longitudinal study has reported that compared to participants who had never smoked, former smoking has no significant impact on gout and active smoking significantly decreases the risk of gout [61]. Remarkably, active smoking presented lower gout risk while former smoking significantly increases gout risk compared to non-smoking [63,64]. Therefore, this inconsistent result might be due to different strategies of smoking status classification.

Similar to active smoking, participants exposed to SHS indicated a higher risk of gout compared to non-smokers after covariate adjustment with time-dependent manner, particularly in participants who were exposed to SHS for more than 31.5 years. The WHO has confirmed that in terms of cardiovascular diseases, the time period for SHS exposure is relatively shorter than for active smoking, whereas long-term effects may take at least one–five years to manifest [29]. The inhalation and exhalation pathways of smoke in active smoking differ from those of SHS. SHS is categorized as side-stream smoke exposure that contains a multitude of toxic agents, generated directly from tobacco products (cigarettes, cigars, and pipes) or exhaled by active smokers or as mainstream smoke [30,65]. A previous study postulated that side-stream smoke contains smaller particles [66] because it is produced in an oxygen-poor environment at lower temperatures, with higher concentrations of ammonia, nitric oxides, and carcinogens [30]. As a result, smaller particles probably penetrate more deeply into the airways when they are inhaled by people exposed to SHS [66]. Importantly, the correlation remains unknown due to no study having been conducted on SHS issues among gout patients. Even so, hyperuricemia is a pivotal risk factor for gout, which was proven to be present among smokers, even though the populations of earlier studies were not categorized into active smokers and those exposed to SHS [14,22,58,67,68]. In line with this situation, we found that 97.5% of gout patients in our study exhibited hyperuricemia. The hypothesis was supported by our data analysis that there was a significant relationship between smoking status and hyperuricemia. As such, the possible elucidation of this phenomenon might suggest that SHS induces a high incidence of hyperuricemia in patients with gout, provoked by a reduction in renal function [67]. Additionally, there is a significant 6.32-fold higher risk of chronic kidney diseases among smokers [59]. 

The risk of reduced renal function is caused by the stimulation of xanthine oxidative stress produced by cigarette smoke [59,68]. In this way, a long duration of smoking produces abundant Reactive Oxygen Species (ROS) [22]. Higher ROS levels induce smooth muscle cell proliferation of renal afferent arterioles and generate further nephrotoxic effects, including a reduction in kidney perfusion and induction of nephrotoxicity, which are induced by smoking-derived oxidative stress, increased serum urate levels, and a consequent increase in the risk of gout [22]. Moreover, a previous study found that SHS exposure for eight weeks enhanced levels of inflammatory cytokines such as IL-17, IL-6, IL-1β, and Tumor Necrosis Factor-α [65]. A previous study also stated that an increased inflammatory response occurred as an event preceding and contributing to the activation of neutrophils in initiating and amplifying monosodium urate (MSU) crystal-induced deposition [69]. Thereby, we suggest that smoking habits, including active smoking and exposure to SHS, induce an escalation of xanthine oxidative stress, which might cause deterioration in renal function that induces hyperuricemia and its consequences of an increased inflammatory response to the initiation and amplification of MSU crystal-induced deposition. This results in an increased risk of gout.

Moreover, our gender data for participants was more significant for females than males. This finding was contradictory to a previous study in which males were dominant among chronic gout patients [70], including in Northern Celebes, Indonesia [8]. In terms of gender issues in this study, it was also reported in the previous study that woman were proven to be the majority of the world’s passive smokers [71]. This might indicate that the contribution of SHS significantly raises the gout disease risk factor. Thus, this finding suggested that a bigger sample size is essential in future studies. Besides, as aforementioned, the SHS participants in this study were subjects who were exposed to at least one active smoker in the home or in public places. Hence, preventing the social impact of SHS is a crucial issue in reducing gout risk.

In addition, it was reported that a high BMI played a critical role in the risk of gout [16,35]. Obesity contributed to hyperinsulinemia with subsequent elevation of urate levels through reduced urinary urate clearance [16,35,61]. This evidence followed an inverse correlation between visceral adiposity and urate clearing rates by the kidneys [35,72]. Therefore, we consider a BMI of >24.9 kg/m^2^ to be a critical factor in increasing the risk of gout, and this should be considered when analyzing the synergistic effects of smoking.

Furthermore, this study found that a novel association of smokers (active smokers and people exposed to SHS) and a BMI >24.9 kg/m^2^ synergistically increased the risk of gout. As aforementioned [27], the combination of smoking and a BMI of >24.9 kg/m^2^ elevated ROS which generated oxidative stress; in particular, excessive adipose tissues in obesity generate proinflammatory cytokines that increase ROS and nitrogen species by macrophages and monocytes and lower antioxidants. Consequently, this creates a worse impact on dysfunction in excreting urate, resulting in the subsequent disequilibrium of resorption of urate, precipitating hyperuricemia and risk of gout. CRP and proinflammatory cytokines, such as IFN-γ, TNF-α, IL-1β, and IL-6, permeate into the bloodstream to cause systemic inflammation. This extends to chronic inflammation present in individuals who are smokers and those with a BMI of >24.9 kg/m^2^ [27,37], leading to hyperuricemia [38]. Taken together, our results suggest that smoking and a BMI of >24.9 kg/m^2^ cause increased ROS, inflammation, and hyperuricemia, which lead to greater risks of gout.

Moreover, a retrospective study revealed that smoking has a positive association with cancer development. Generally, the possible mechanism of the tobacco smoke’s effect is somatic gene mutation and immunosuppression, such as a decrease in the responsiveness of T cells [73]. Another important issue is that smoking habits are the most preventable cause of increased cardiovascular disease risk and sudden death [74], since smoking produces oxidative processes, negatively affecting platelet function, fibrinolysis, inflammation and vasomotor function [75]. Thus, preventing smoking habits is an important issue to raise in prevention strategies against cancer and cardiovascular disease, which are related to gout.

Our study has several strengths. To the best of our knowledge, this is the first study that assessed novel issues regarding smoking status (active smokers and people exposed to SHS). We also evaluated detailed information on time and dose, which encompassed the duration (in years) and the average number of cigarettes consumed per day by active smokers, and the number of cigarettes per day to which people were exposed to as SHS. This was accompanied by another novel finding of the synergistic effect of smokers (active smokers and people exposed to SHS) and a BMI of >24.9 kg/m^2^, significantly elevating the risk of gout. Moreover, this was a community-based study with stratified multistage cluster sampling. Thus, the findings might be generalizable to all Indonesians. 

Along with its strengths, this study also had some limitations. The study was conducted using a retrospective case-control design, which limits the establishment of one-directional causality between smoking status and the risk of gout disease. However, the inclusion of patients with gout was appropriate, since we solely focused on newly diagnosed gout participants to analyze risk factors, which benefitted a retrospective estimation of one-directional causality between risk factors and gout. Smoking status including SHS was assessed by self-administered questionnaires; this might be prone to recall bias data. However, we utilized a validated and reliable questionnaire to overcome this kind of limitation. Still, we excluded former smokers in the analysis due to their small number. These limitations should be considered when interpreting and applying the results of this study.

## 5. Conclusions

Both active smoking and SHS were suggested to increase gout risk in a time-dependent manner. A dose-dependent relationship to increases gout risk was also found in active smokers. The synergic effect between smoking (active smokers and exposure to SHS) and a BMI of >24.9 kg/m^2^ escalated gout risk among Indonesians. Combining this study result and the fact that Indonesia is one of the top five countries in the world for tobacco use, smoking habits among communities in Indonesia need the community health nurses’ attention. These health professionals need to educate individuals to protect themselves from smoking exposure in order to prevent gout.

## Figures and Tables

**Table 1:** Distributions of Demographic Characteristics Between Healthy Controls and Patients with Gout (*N* = 385).

Characteristics	Healthy Control Group *n* (%)	Gout Group *n* (%)	*p* Value
Gender			<0.05
Female	212 (69.70%)	47 (58%)	
Male	92 (30.30%)	34 (42%)	
Marital status			
Unmarried	70 (23%)	15 (18.50%)	0.38
Married	234 (77%)	66 (81.50%)	
Educational level			
College	61 (20%)	13 (16%)	<0.05
Senior high school	144 (47.40%)	27 (33.30%)	
Junior high school or below	99 (32.60%)	41 (50.70%)	
Monthly income			
>US$214	40 (13.10%)	12 (14.80%)	0.68
US$128~214	68 (22.40%)	21 (25.90%)	
<US$128	196 (64.50%)	48 (59.30%)	
Occupational status			0.35
Employed	151 (49.70%)	45 (55.60%)	
Unemployed	153 (50.30%)	36 (44.40%)	
Age (years)			<0.05
≤47	164 (53.90%)	34 (42%)	
>47	140 (46.10%)	47 (58%)	
Uric acid level (mg/dL)			<0.01
≤5.7	195 (64.10%)	2 (2.50%)	
>5.7	109 (35.90%)	79 (97.50%)	
Systolic blood pressure (mmHg)			
≤131	162 (53.30%)	31 (38.30%)	<0.05
>131	142 (46.70%)	50 (61.70%)	
Diastolic blood pressure (mmHg)			
≤80	152 (50%)	44 (54.30%)	0.48
>80	152 (50%)	37 (45.70%)	
BMI (kg/m^2^)			<0.05
≤24.9	151 (49.70%)	22 (27.20%)	
>24.9	153 (50.30%)	59 (72.80%)	
Alcohol Consumption			0.001
Non-drinker	253 (65.70%)	52 (13.50%)	
Drinker	51 (13.20%)	29 (7.50%)	

Note: The *X*^2^ test was used to compare between groups; SHS, second-hand smoke; pcs = pieces.

**Table 2:** Distributions of Potential Risk Factors Between Healthy Controls and Patients with Gout (*N* = 385).

Characteristics	Healthy Control Group *n* (%)	Gout Group *n* (%)	*p* Value
Smoking status	304 (100%)	81 (100%)	
Non-smoker	212 (69.70%)	22 (27.20%)	<0.01
Exposed to SHS	47 (15.50%)	35 (43.20%)	
Active smoker	45 (14.80%)	24 (29.60%)	
No. of cigarettes consumed per day by an active smoker (pcs)	257 (100%)	46 (100%)	
0	212 (82.50%)	22 (47.80%)	<0.01
>0~≤16	25 (9.70%)	13 (28.30%)	
>16	20 (7.80%)	11 (23.90%)	
Duration active smoker (years)	257 (100%)	46 (100%)	
0	212 (82.50%)	22 (47.80%)	
>0~≤23	24 (9.30%)	11 (23.90%)	<0.01
>23	21 (8.20%)	13 (28.30%)	
Pack-year of active smoker	257 (100%)	46 (100%)	
0	212 (82.50%)	22 (47.80%)	
>0~<20	28 (10.90%)	10 (21.70%)	<0.01
>20	17 (6.60%)	14 (30.50%)	
No. of cigarettes per day generating SHS exposure (pcs)	259 (100%)	57 (100%)	
0	212 (81.8%)	22 (38.6%)	<0.01
>0~≤20	38 (14.7%)	24 (42.1%)	
>20	9 (3.5%)	11 (19.3%)	
Duration of exposure to SHS (years)	259 (100%)	57 (100%)	
0	212 (81.8%)	22 (38.6%)	
>0~≤31.50	30 (11.6%)	11 (19.3%)	<0.01
>31.50	17 (6.6%)	24 (42.1%)	
Pack-year of SHS exposure	259 (100%)	57 (100%)	
0	212 (81.90%)	22 (38.60%)	
>0~≤26.5	31 (12.00%)	10 (17.5%)	<0.01
>26.5	16 (6.10%)	25 (43.9%)	

Note: The *X*^2^ test was used to compare between groups; SHS, second-hand smoke; pcs = pieces.

**Table 3:** Odds Ratio (OR) and Adjusted Odds Ratio (AOR) of The Risk of Gout (*N* = 385).

Characteristic	Healthy Control Group *n* (%)	Gout Group *n* (%)	OR (95% CI)	AOR (95% CI)
Smoking status	304 (100%)	81 (100%)		
Non-smoker	212 (69.70%)	22 (27.20%)	1.00	1.00
Exposed to SHS	47 (15.50%)	35 (43.20%)	7.17 (3.86~13.33) **	4.67 (2.18~10.00) **
Active smoker	45 (14.80%)	24 (29.60%)	5.13 (2.65~9.96) **	3.26 (1.07~9.90) *
Duration as active smoker (years)	257 (100%)	46 (100%)		
0	212 (82.50%)	22 (47.80%)	1.00	1.00
>0~≤23	24 (9.30%)	11 (23.90%)	2.93 (1.13~7.60) *	2.34 (0.46~11.98)
>23	21 (8.20%)	13 (28.30%)	7.45 (3.45~16.08) **	5.95 (1.41~25.03) *
No. of cigarettes consumed per day by an active smoker	257 (100%)	46 (100%)		
0	212 (82.50%)	22 (47.80%)	1.00	1.00
>0~≤16	25 (9.70%)	13 (28.30%)	2.83 (0.95~8.42)	2.50 (0.45~13.96)
>16	20 (7.80%)	11 (23.90%)	6.54 (3.15~13.56) **	5.15 (1.28~20.63) *
Pack~year of active smoker	257 (100%)	46 (100%)		
0	212 (82.50%)	22 (47.80%)	1.00	1.00
>0~<20	28 (10.90%)	10 (21.70%)	3.44 (1.47~8.01) **	2.94 (0.68~12.76)
>20	17 (6.60%)	14 (30.50%)	7.93 (3.45~18.24) **	7.18 (1.53~33.67) *
Duration of exposure to SHS (years)	259 (100%)	57 (100%)		
0	212 (81.80%)	22 (38.60%)	1.00	1.00
>0~≤31.5	30 (11.60%)	11 (19.30%)	3.53 (1.55~8.01) **	2.11 (0.79~5.66)
>31.5	17 (6.60%)	24 (42.10%)	13.60 (6.35~29.11) **	10.12 (3.51~29.14) **
No. of cigarettes per day generating SHS exposure	259 (100%)	57 (100%)		
0	212 (81.08%)	22 (38.60%)	1.00	1.00
>0~≤20	38 (14.70%)	24 (42.10%)	6.08 (3.10~11.95) **	4.53 (1.89~10.84) **
>20	9 (3.50%)	11 (19.30%)	11.77 (4.40~31.51) **	4.37 (1.33~14.28) **
Pack-year of SHS exposure	259 (100%)	57 (100%)		
0	212 (81.90%)	22 (38.60%)	1.00	1.00
>0~≤26.5	31 (12.00%)	10 (17.5%)	3.10 (1.34~7.18) **	1.66 (0.58~4.72)
>26.5	16 (6.10%)	25 (43.9%)	15.06 (7.00~32.38)	9.95 (3.64~27.22) **

Note: BMI: body-mass index; SHS: second-hand smoke. The OR was calculated with a binary logistic regression test. The AOR was calculated by a multiple logistic regression test and adjusted for alcohol consumption, gender, educational level, age, urate levels, systolic blood pressure, and body-mass index. * Indicates a significant difference in values between groups at *p* < 0.05; ** Indicates a significant difference in values between groups *p* < 0.01.

**Table 4:** Synergistic effect between BMI and smoking status on the risk of gout (*N* = 385).

Characteristics	Healthy Control Group (*n* = 304) (%)	Gout Group (*n* = 81) (%)	OR (95% CI)	AOR (95% CI)
BMI ≤24.9 kg/m^2^ and non-smoker	106 (34.90%)	6 (7.40%)	1.00	1.00
BMI ≤24.9 kg/m^2^ and smoker (active smoker and exposed to SHS)	45 (14.80%)	16 (19.80%)	6.28 (2.31~17.09) **	3.18 (1.01~9.97) *
BMI >24.9 kg/m^2^ and non-smoker	106 (34.90%)	16 (19.80%)	2.67 (1.01~7.08) *	1.94 (0.66~5.64)
BMI >24.9 kg/m^2^ and smoker (active smoker and exposed to SHS)	47 (15.40%)	43 (53.10%)	16.16 (6.43~40.58) **	9.65 (3.25~28.65) **

Note: BMI: Body Mass Index; OR: odds ratio; AOR: adjusted odds ratio; CI: confidence interval; SHS: second-hand smoke. OR was calculated with a binary logistic regression test. AOR was calculated with a multiple logistic regression test adjusted for alcohol consumption, gender, educational level, age, urate levels, systolic blood pressure, and body-mass index. * Indicates a significant difference in values between groups at *p* < 0.05; ** Indicates a significant difference in values between groups at *p* < 0.01.

**Table 5:** Odds ratio (OR) and adjusted odds ratio (AOR) for the risk of hyperuricemia (*N* = 381).

Characteristic	Norm Uricemia (<6 mg/dL) *n* (%)	Hyperuricemia (>6 mg/dL) *n* (%)	OR (95% CI)	AOR (95% CI)
Smoking status	227 (100%)	154 (100%)		
Non-smoker	163 (71.8%)	69 (44.8%)	1.00	1.00
Exposed to SHS	32 (14.1%)	49 (31.8%)	3.58 (2.12~6.05) **	3.37 (1.93~5.89) **
Active smoker	32 (14.1%)	36 (23.4%)	2.65 (1.53~4.59) **	1.39 (0.63~3.07)

Note: Four patients who underwent urate lowering therapy were excluded. OR: odds ratio; AOR: adjusted odds ratio; CI: confidence interval; SHS: second-hand smoke. OR was calculated with a binary logistic regression test. AOR was calculated with a multiple logistic regression test adjusted for alcohol consumption, gender, educational level, age, systolic blood pressure, and body-mass index. ** Indicates a significant difference in values between groups at *p* < 0.01.

## Data Availability

Data sharing is not applicable to this article.
